# Chemical Profiling and Biological Activities of *Pelargonium graveolens* Essential Oils at Three Different Phenological Stages

**DOI:** 10.3390/plants11172226

**Published:** 2022-08-27

**Authors:** Samiah Hamad Al-Mijalli, Hanae Naceiri Mrabti, Hamza Assaggaf, Ammar A. Attar, Munerah Hamed, Aicha EL Baaboua, Nasreddine El Omari, Naoual El Menyiy, Zakaria Hazzoumi, Ryan A Sheikh, Gokhan Zengin, Stefania Sut, Stefano Dall’Acqua, Abdelhakim Bouyahya

**Affiliations:** 1 Department of Biology, College of Sciences, Princess Nourah Bint Abdulrahman University, Riyadh 11671, Saudi Arabia; 2 Laboratory of Pharmacology and Toxicology, Bio Pharmaceutical and Toxicological Analysis Research Team, Faculty of Medicine and Pharmacy, Mohammed V University in Rabat, Rabat 10000, Morocco; 3 Department of Laboratory Medicine, Faculty of Applied Medical Sciences, Umm Al-Qura University, Makkah 21955, Saudi Arabia; 4 Department of Pathology, Faculty of Medicine, Umm Al-Qura University, Makkah 21955, Saudi Arabia; 5 Biology and Health Laboratory, Department of Biology, Faculty of Science, Abdelmalek-Essaadi University, Tetouan 93000, Morocco; 6 Laboratory of Histology, Embryology, and Cytogenetic, Faculty of Medicine and Pharmacy, Mohammed V University in Rabat, Rabat 10000, Morocco; 7 Laboratory of Pharmacology, National Agency of Medicinal and Aromatic Plants, Taounate 34025, Morocco; 8 Plant and Microbial Biotechnology-Moroccan Foundation for Advanced Science, Innovation and Research, Rabat 10100, Morocco; 9 Biochemistry Department, Faculty of Science, King Abdulaziz University, Jeddah 21589, Saudi Arabia; 10 Department of Biology, Science Faculty, Selcuk University, Konya 42130, Turkey; 11 Department of Pharmaceutical and Pharmacological Sciences, University of Padova, Via Marzolo 5, 35131 Padova, Italy; 12 Laboratory of Human Pathologies Biology, Department of Biology, Faculty of Sciences, Mohammed V University in Rabat, Rabat 10000, Morocco

**Keywords:** *Pelargonium graveolens*, essential oils, antidiabetic, anti-inflammatory, antibacterial

## Abstract

The aim of this work was the determination of *Pelargonium graveolens* (aerial parts) volatile compounds at three developmental stages and the evaluation of their antioxidant, antidiabetic, dermaprotective, anti-inflammatory, and antibacterial effects. The aerial parts of *Pelargonium graveolens* were collected at three stages, namely the vegetative, beginning, and full flowering. *Pelargonium graveolens* essential oils were extracted from the dried materials of these aerial parts by hydrodistillation. The volatiles were analyzed by Gas Chromatography-Mass Spectrometry GC-MS, and the antioxidant activity was assessed by DPPH, ABTS, H_2_O_2_, and FRAP assays. The in vitro antidiabetic effect was evaluated by the inhibition of α-amylase, α-glucosidase, and lipase enzymes, while the antibacterial activity was assessed against six bacterial strains using an agar well diffusion assay and a microdilution method. The main constituents were menthol, menthene, eremophilene, isoborneol, isogeraniol, α-pinene, linalyl acetate, and 3-carene, with quantitative differences at the three phenological stages. The essential oil at the full flowering stage showed the best antioxidant activity, with IC_50_ values of 83.26 ± 0.01, 116.42 ± 0.07, 132.25 ± 0.11, and 48.67 ± 0.04 μg/mL for DPPH, FRAP, ABTS, and H_2_O_2_ assays, respectively. This oil also exhibited significant effects against α-amylase (IC_50_ = 43.33 ± 0.01 μg/mL), α-glucosidase (IC_50_ = 19.04 ± 0.01 μg/mL), lipase (IC_50_ = 24.33 ± 0.05 μg/mL), 5-lipoxygenase (IC_50_ = 39.31 ± 0.01 μg/mL), and tyrosinase (IC_50_ = 124.49 ± 0.07 μg/mL). The essential oil extracted at the full flowering stage showed the best antibacterial effect against a panel of microorganisms with diameter inhibition zones ranging between 11.00 ± 0.17 mm and 17.30 ± 0.17 mm and MIC values from 0.25% to 2% *v*/*v*. Overall, the results presented here suggest that the full flowering stage is the best optimal harvest time of *Pelargonium graveolens* for food and pharmaceutical applications.

## 1. Introduction

In recent years, the general public has shown an increased interest in the use of medicinal plants in preference to synthetic drugs due to their potential as a source of natural molecules often devoid of side effects and that can be obtained at a lower cost [1,2]. Among the different types of these natural substances, plant secondary metabolites, including volatile substances or essential oils (EOs) [3,4,5,6,7], phenolic compounds and flavonoids are of particular interest because of their health benefits [8]. Today, the essential oils of various aromatic plants and their derivatives have been the subject of several studies due to their various uses other than the classic roles of raw material in different industries such as pharmaceutical, agronomic, food, health, cosmetic, and perfumery [9]. Moreover, several researchers have reported a high variation in the yield and/or chemical composition of essential oils, as well as their potential biological proprieties [10,11,12,13,14]. This variation mainly depended on several factors such as geographical region, environmental conditions, cultivars, distillation method, storage and processing conditions, post-harvest storage, transplanting date, and intercropping [15]. The genus *Geranium*, known as rose-scented geranium, is an important and valuable aromatic crop belonging to the Geraniaceae family, commonly cultivated for valuable volatile oil production. Out of 25 *Pelargonium* species, only four species (*Pelargonium graveolens*, *Pelargonium odoratissium*, *Pelargonium capitatum*, and *Pelargonium radens*) are used in the production of essential oils [16].

*Pelargonium graveolens* (*P. graveolens*) L’Herit is an aromatic, hairy herbaceous shrub, reaching 1.3 m high and 1 m wide. The leaves are prickly and carved. The flowers are small, usually pink. *P. graveolens* (geranium) is native to southern Africa and is widely cultivated in several countries, mainly in Russia, Egypt, Algeria, Morocco, Congo, Japan, and some continents such as Central America and Europe [17]. Among several *P. graveolens* extracts that may be useful as bioactive natural plant products; the essential oil was reported to possess a wide range of biological and pharmacological properties such as antioxidant [2,18], antibacterial [15,19], antifungal [20], hypoglycemic [21], anti-inflammatory [22], and anticancer [16]. These activities could be related to the presence of some bioactive compounds of *P. graveolens* essential oils (PGEOs), including citronellol, geraniol, and linalool as major compounds [23]. Accordingly, the investigation of the chemical composition of this plant has become recommended in herbal medicine. Thus, it is important to establish the scientific basis of their therapeutic actions, which can serve as a source for the development of effective and harmless drugs. Therefore, this study was conducted to (i) analyze the chemical composition of Moroccan PGEO at three different developmental stages (vegetative, beginning, and full flowering stages), (ii) evaluate their antioxidant, antibacterial, and anti-diabetic effects, and (iii) investigate the acetylcholinesterase and tyrosinase inhibitory activities of three EOs in order to correlate the pharmacological activities and PGEO compounds.

## 2. Results & Discussion

### 2.1. Chemical Composition

The chemical composition of *P. graveolens* at three phenological stages was determined by the gas chromatography–mass spectrometry (GC-MS) method. The results obtained are listed in Table 1. Fifty-two compounds were identified at the vegetative stage, fifty-one compounds at the beginning flowering stage, and fifty-five elements at the full flowering stage. Concerning the PGEO at the vegetative stage, the total of the identified compounds was 92.98, divided into three classes: monoterpene hydrocarbons (20.84%), oxygenated monoterpenes (39.08%), and sesquiterpene hydrocarbons (25.41%). For the PGEOs at the beginning stage, the percentages of monoterpene hydrocarbons, oxygenated monoterpenes, and sesquiterpene hydrocarbons were 18.63%, 35.1%, and 30.19%, respectively. Similar results were found with PGEO, which revealed 17.99% monoterpene hydrocarbons, 41.31% oxygenated monoterpenes, and 24.25% sesquiterpene hydrocarbons.

The main components identified in PGEOs at the three phenological stages were menthol (15.80%, 14.06%, and 20.57%), isogeraniol (15.47%, 15.05%, and 9.14%), eremophilene (8.34%, 9.02%, and 8.19%), and menthene (7.87%, 6.70%, and 9.97%) at the vegetative, beginning, and end of flowering stages, respectively. Other constituents of essential oils were present in small quantities compared to the aforementioned compound. The percentage of menthol was very high at the full flowering stage (20.57%) compared to the vegetative stage (15.80%) and at the beginning flowering stage (14.06%). Moreover, the isogeraniol level was important at the vegetative stage (15.47%), which confirms that harvesting at different growth stages has a considerable effect on the composition of EOs.

Previous works have reported the PGEO chemical composition [9,10,19,23,24,25,26,27,28,29], but large differences were present, especially in essential oils obtained from plants grown in different countries. These can be explained by different climatic and environmental conditions but also by different distillation procedures and methods. Iran’s PGEO was dominated by citronellol (48.44%), octen-1-ol (18.61%), and geraniol (9.70%) [28]. According to the results obtained by Boukhris et al. [17], Tunisian PGEOs contained high levels of *β*-citronellol (21.9%), citronellyl formate (13.2%), and geraniol (11.1%). However, an analysis of PGEOs from Serbia revealed that citronellol (24.54%), geraniol (15.33%), citronellyl formate (10.66%), and linalool (9.80%) were the major components [25]. Lalli et al. [30] reported that in Johannesburg, South Africa, the main components of PGEO were isomenthone (84.0%), methone (2.8%), and myrcene (0.9%). Egyptian geranium EO was dominated by citronellol (29.90%), trans-geraniol (18.03%), and 10-epi-γ-eudesmol (8.27%) [16].

### 2.2. Antibacterial Activity

#### 2.2.1. Disc Diffusion Method

The in vitro antibacterial activity of PGEO at three different developmental stages (vegetative, beginning, and full flowering stages) was first investigated using a disk diffusion assay against reference pathogenic bacteria (Gram-positive and Gram-negative), and the results are presented in Table 2. The values obtained from the inhibition zones, expressed in millimetres, showed a variation in the results according to the strains and the examined stages of PGEO. This oil at the beginning and full flowering stages showed vigorous antimicrobial impact against all tested strains compared to the vegetative stage (the highest recorded diameter of this last was 17.30 ± 0.17 mm at the full flowering stage of PGEO) with the exception of *Salmonella typhimurium* (*S.* *typhimurium*) ATCC700408.

The measured inhibition zones of PGEO were classified between sensitive effect (9–14 mm) to very sensitive effect (15–19 mm) according to the scale proposed by Moreira et al. [31], in which *Escherchia coli* (*E. coli*) ATCC 25922, *Proteus mirabilis* (*P. mirabilis)* ATCC 25933, *Bacillus subtilis* (*B. subtilis*) ATCC 6633, *Staphylococcus aureus* (*S. aureus*) ATCC 29213, and *Listeria monocytogenes (L. monocytogenes*) ATCC 13932 were the most sensible strains. However, *S.* *typhimurium* ATCC 700408 was resistant to PGEO since the bacterial agent exhibited the smallest inhibition diameter of PGEO at the vegetative stage and was sensitive at the other two flowering stages (Table 2).

#### 2.2.2. MIC and MBC Determination

The quantitative effect of PGEO at three developmental stages was assessed using the microdilution technique for bacterial isolates. The minimal inhibitory concentration (MIC) values found are summarized in Table 3. Similar to the disk diffusion test outcomes, PGEOs at both flowering phases recorded higher inhibitory concentrations than PGEOs at the vegetative stage on all microorganisms tested (MICs of 0.12 % (*v*/*v*) and 0.5 % (*v*/*v*), respectively). To illustrate this, *B. subtilis* ATCC 6633, *L. monocytogenes* ATCC 13932, and *S. aureus* ATCC 29213 noted the lowest MIC values of PGEO (0.25 % (*v*/*v*)) at the full flowering stage, followed by *E. coli* ATCC 25922 and *P. mirabilis* ATCC 25933 with MICs equal to 0.5% and 1% (*v*/*v*), respectively, whereas 2% (*v*/*v*) was the highest MIC of PGEO obtained for *S. typhimurium* ATCC 700408 (Table 3).

The MBC measurement showed a bactericidal effect of PGEO at the full flowering stage against *L. monocytogenes* ATCC 13932, *P. mirabilis* ATCC 25933, and *S. typhimurium* ATCC 700408 with MBCs of 0.25%, 1%, and 2%, respectively. Other strains have shown a double effective dose to kill primarily *E. coli* ATCC 25922, *B. subtilis* ATCC 6633, *S. aureus* ATCC 29213 at both the beginning and full flowering stages of PGEO (Table 3). It should be noted that all the strains tested were sensible to chloramphenicol, used as a positive control, by both techniques used (Table 2 and Table 3), except *S. typhimurium* ATCC 700408 and *B. subtilis* ATCC 6633, following the guidelines provided by the EFSA and EUCAST [32,33].

The pathogenic bacteria are becoming increasingly resistant to antibiotics, challenging the medical industry to treat microbial infections with the most effective antibiotic classes [34,35]. However, aromatic and medicinal plant species are extensively used in traditional medicine, and they have been reported as suitable sources of rich natural alternative compounds for pharmacological purposes.

Numerous studies on essential oils extracted from aromatic and medicinal plants have demonstrated their inhibitory action against the most infectious bacterial agents [36,37]. Indeed, Gram-positive bacteria are more sensitive to essential oils than Gram-negative bacteria [38,39]. In the present work, PGEO extracted at the full flowering stage exhibited a notable antibacterial activity against all of the tested bacterial strains, both Gram-negative and Gram-positive, by disk diffusion assay (inhibitory diameter was between 11.00 ± 0.17 mm and 17.30 ± 0.17 mm) and broth microdilution technique (MIC values between 0.25% (*v*/*v*) and 1% (*v*/*v*)), with the exception of *S.* *typhimurium* ATCC 700408 (Table 2 and Table 3).

Likewise, our findings were consistent with those obtained in previous Tunisian research conducted by Hsouna and Hamdi [19]. The authors demonstrated from the comparison of EOs and organic extracts of *P. graveolens*, that all extracts were active against *B. subtilis* ATCC 6633 (22 ± 0.0 mm) and S. aureus ATCC 25923 (23 ± 0.3 mm), in particular PGEO [19]. P. mirabilis was also sensitive to PGEOs obtained from the floral parts with an MIC value (1% (*v*/*v*)) and MBC amount (2% (*v*/*v*)) equal to those endorsed in our conclusions [8]. Furthermore, the same authors revealed the significant antibacterial activity of PGEOs extracted from the leaves and flowers on *S. typhimurium* NCTC 6017 [8]. Their findings enabled them to conclude that the EOs of the floral organ have a higher activity (inhibition diameter of 16 ± 1.5 mm, MIC of 2% (*v*/*v*), and MBC equal to 5% (*v*/*v*)) than that observed in the leaf organ of P. graveolens (inhibition diameter of 14 ± 1.5 mm and MIC of 10%). These outcomes are in agreement with those obtained in our study.

Previous data on the antibacterial activity of PGEOs report some discrepancies [9,19,40,41]. Fluctuations were cited in the work of Ghannadi et al. [9] on *E. coli* (PTCC 1330) resistant to PGEO (9.5 ± 0.7 mm vs. 13.6 mm at full flowering stage). Two other research articles have been published on the antibacterial properties of PGEOs from the Fez-Meknes and Souss-Massa regions in Morocco. From P. graveolens aerial parts, Sadiki et al. [41] revealed that the EOs have an equal inhibitory and bactericidal impact on *E. coli* and *S. aureus* clinical isolates from the Military hospital of Moulay Ismail in Morocco at MICs of 1.12% (*v*/*v*) and 2.5% (*v*/*v*), for both strains, respectively. El Asbahani et al. [40] found a higher antimicrobial potency (MICs ranging between 1.4 and 2.8 μg/mL) than that obtained in our work on *E. coli*, *S. typhimurium*, *L. monocytogenes*, and *S. aureus*.

Ghannadi et al. [9] and Hsouna and Hamdi [19] reported that PGEO derived from Iran and Tunisia had no effect against a foodborne reference strain of *L. monocytogenes* PTCC 1297 and *L. monocytogenes* 12228, respectively. On the other hand, the Moroccan PGEOs inhibit the growth of *L. monocytogenes* (17.30 ± 0.17 mm) with an MIC value of 0.25% (*v*/*v*) equal to MBC (Table 2). The data obtained in this work revealed that the Moroccan PGEO had the lowest values against *B. subtilis* ATCC 6633 (MIC = 0.25% (*v*/*v*) and MBC = 0.5% (*v*/*v*)) the differently high MIC and MBC reported by Hsouna and Hamdi, [19].

In this work, the biological activities noted, considering the differences at the three developmental stages, could be attributed to the EO chemical composition. At the end of the flowering stage, the PGEO showed higher levels of menthol (20.57%), menthene (9.97%), and isogeraniol (9.14%) than those obtained in the other two phases (15.80% of menthol, 15.47% of isogeraniol, and 7.87% of menthene at the vegetative phase vs. 15.05% of isogeraniol, 14.06% of menthol, and 6.70% of menthene at the beginning of the flowering phase) (Table 1). These findings are identical to those of Bouyahya et al. [42] and Boukhris et al. [43] when they studied the correlation between phenological changes in the EO of medicinal plants. Moroccan PGEO contains menthol as the main chemotype, and its activity against *S. aureus* and *E. coli* can be explained by the effect of (+)-menthol on membrane permeability through the perturbation of the lipid fraction of the bacterial plasma membrane [44]. Consequently, full-stage PGEOs showed significant antibacterial performance compared to chloramphenicol in the case of resistant *S. typhimurium* and *B. subtilis*.

Indeed, antibacterial agents may act by increasing membrane permeability and decreasing membrane integrity, blocking oxidative respiration, and inhibiting DNA and protein synthesis. Interestingly, recent investigations have shown that certain bacterial strains can resist these antibacterial agent mechanisms through a microbial cell-to-cell communication system called quorum sensing [37,42].

Recently, different combinations of EO or EOs-antibiotics have become a new trend for investigating possible synergistic interactions. The research team led by Kafa et al. [45] showed synergistic activity between PGEO and colistin, with a fractional inhibitory concentration index of less than 0.5% against drug-resistant *Acinetobacter baumannii* (*A*. *baumannii*) clinical isolates. Likewise, the effectiveness of this combination was not limited to the reduction in the bacterial population (2–32 fold) but also from 48% to 90% to the elimination of the biofilm layer of the *A. baumannii* tested strains [45].

Until now, little has been known about the synergy of menthol, menthene, and isogeraniol, and these associations require further investigation to better elucidate their mechanisms of action at different levels (cellular, sub-cellular, and molecular) and also their safety; alone or in combination, which could make them candidates or lead compounds for the development of natural antibiotics.

### 2.3. Antioxidant Activity

The antioxidant potency screening of PGEOs at three developmental stages was performed using 2,2-diphenyl-1-picrylhydrazy (DPPH), Frequent Rapid Activity Period (FRAP), 2,2′-Azinobis-(3-Ethylbenzthiazolin-6-Sulfonic Acid (ABTS), and H_2_O_2_ assays, and the results are presented in Table 4. The IC_50_ value of PGEOs showed a marked increase in the antioxidant effect from dormancy, the beginning to full flowering phases in all protocols. Undoubtedly, the best-estimated proton reduction activity of PGEOs was significantly observed at the full flowering stage (*p* < 0.05) and can be classified according to IC_50_ values as follows; H_2_O_2_ (48.67 ± 0.04 μg/mL), DPPH (83.26 ± 0.01 μg/mL), FRAP (116.42 ± 0.07 μg/mL), and ABTS (132.25 ± 0.11 μg/mL).

During the vegetative time, PGEO revealed the highest IC_50_ compared to the other stages. Ascorbic acid, used as a positive control, showed the highest anti-radical effect compared to PGEO at the full flowering stage using DPPH (IC_50_ = 15.13 ± 0.04 μg/mL), H_2_O_2_ (IC_50_ = 23.34 ± 0.03 μg/mL), FRAP (IC_50_ = 31.47 ± 1.07 μg/mL), and ABTS (IC_50_ = 42.22 ± 0.05 μg/mL) methods (Table 4).

Due to the complexity and great diversity in the chemical composition of essential oils, many protocols were established to assess their free radical scavenging activity [42,43]. The results indicated that PGEO has a higher antioxidant capacity in the full flowering phase compared to PGEOs at the beginning flowering and dormancy phases, respectively. This interesting antioxidant activity is consistent with those collected by various surveys [2,18,43,46].

To our knowledge, there is only one Tunisian research investigation carried out by Boukhris et al. [43], who evaluated the antioxidant impact of PGEO at three developmental phases. They measured a higher DPPH antioxidant power for an IC_50_ value of 1 ± 0.01 g/mL compared to that verified by ABTS analysis (7 ± 0.015 mM trolox equivalent) at the full flowering stage [43]. Our results are in agreement with those obtained by Boukhris et al. [43] and Ćavar and Maksimović, [2], who also confirmed this antioxidant capacity using the DPPH test. It is noted that no scientific exploration has measured the antioxidant capacity of PGEO using the H_2_O_2_ assay.

Considering the antiradical scavenging activity and the EO constituents, we can postulate the transfer of the proton–electron pair of the free radical scavenger on the reactive nitrogen and oxygen species. We can consider the scavenger monoterpene as limonene α-terpineol, carvone, eucalyptol, and menthol, as previously reported [47]. The scavenging activity reduces the radical accumulation and therefore protects the cells from damage. The use of these natural antioxidants specifically targeting the generation of reactive oxygen species will avoid cellular oxidative stress and therefore constitute molecular building blocks in the screening of antioxidant substances used in the prevention of multiple pathologies.

### 2.4. Anti-Diabetic Activity

The inhibition of carbohydrate and glyceride hydrolyzing enzymes is a promising therapeutic strategy in the management of type 2 diabetes mellitus (T2DM). In our case, we evaluated in vitro the inhibitory capacity of EOs, obtained at different phenological stages, on the catalytic activity of α-glucosidase, α-amylase, and lipase. The results showed that the EO of the full flowering stage presents the best values against α-glucosidase (IC_50_ = 19.04 ± 0.01), lipase (IC_50_ = 24.33 ± 0.05), and α-amylase (IC_50_ = 43.33 ± 0.01) compared to the reference compounds (Table 5). The antidiabetic effect of *P. graveolens* plant extracts and EOs were previously reported in vivo models. Boukhris et al. [21] were among the first researchers to investigate this effect by administering two doses (75 and 150 mg/kg b.w.) of PGEO to alloxan-induced diabetic rats and, after one month of treatment, observed a decrease in blood glucose levels compared to glibenclamide (600 μg/kg b.w.), an antidiabetic drug. The antidiabetic effect was also demonstrated in the same animal model but with intraperitoneal (42.5 mg/kg) EO administration [48]. Fadwa et al. [49] examined the anti-hyperglycemic effect of *P. graveolens* leaf aqueous extract (PGLAE) on streptozotocin-induced diabetic rats. After repeated oral administration for 15 days, 40 mg/kg of the extract significantly reduced blood glucose levels in diabetic animals. These effects have been found to be attributed to the plant’s ability to induce the expansion of β-cell mass, inhibit carbohydrate absorption, and increase acute insulin production [50], but a very different composition is expected from PGLAE and the PGEO. On the other hand, other in vitro studies have targeted the inhibition of digestive enzymes (α-amylase and α-glucosidase) to assess the antidiabetic activity of *P. graveolens* [8,51,52]. Initially, Afifi and co-workers showed that PGLAE inhibits the activity of these enzymes with an IC_50_ of 4.6 ± 0.1 mg/mL [51]. Moreover, in 2020, a Tunisian research team found that the inhibitory potency of methanolic leaf extracts of *P. graveolens* was the highest (14.33 ± 0.09 mg ACE/g), followed by ethanolic extracts (12.21 ± 0.1 mg ACE/g), and EOs (5.83 ± 0.55 mg ACE/g) against α-amylase [8]. Ahamad and Uthirapathy [52] used EOs from the leaves of this plant that showed an IC_50_ value of 93.72 ± 4.76 μg/mL against the α-glucosidase enzyme, compared to acarbose (IC_50_ = 80.4 ± 2.17 μg/mL). α-pinene and menthol were among the active compounds. In fact, daily doses of menthol (25, 50, and 100 mg/kg b.w.) decreased hepatic glycogen, plasma insulin, and total hemoglobin levels, with decreased glycosylated hemoglobin, blood glucose levels, and alterations in hepatic glucose metabolic enzymes in diabetic animals; this was explained by the ability of menthol to inhibit pancreatic β-cell apoptosis [53]. Likewise, α-pinene was able to reduce fasting blood glucose levels in alloxan-induced diabetic mice 2 and 24 h after the treatment [54].

### 2.5. Anti-Inflammatory Activity

Chronic inflammatory diseases can be associated with microbial infections (bacteria, fungi, virus, and parasites) as well as certain complex diseases such as cancer, cardiovascular diseases, and neurodegenerative pathologies. Reducing inflammation is a crucial way to reduce the complications of these pathologies, as well as their prevention. In this context, different pharmacological investigations have focused on the screening of anti-inflammatory agents from natural resources [55].

In this work, we evaluated the anti-inflammatory activity of PGEO at three phrenological stages by testing its ability to inhibit the activity of lipoxygenase, an enzyme catalyzing fatty acid oxidation and responsible for the production of leukotrienes, the major mediators of inflammation. Our results showed that PGEO at the full flowering phase exhibits the best inhibitory activity (IC_50_ = 39.31 ± 0.01 μg/mL) compared to quercetin (IC_50_ = 24.89 ± 0.02 μg/mL); which corroborates the results obtained in the previous studies.

Indeed, this was verified in 2015 when Ghanizadeh and colleagues induced ulcerative colitis using acetic acid in rats to record at the end of their experiment a significant anti-inflammatory effect of *P. graveolens* methanolic extract (100 mg/kg) on this colitis [56], but the chemical composition of PGEO is probably very different. With the same experimental model, Bastani et al. [22] reported anti-inflammatory effects with PGEO (100, 200, and 400 mg/kg, p.o.). Additionally, Boukhatem et al. [57] recently revealed an important anti-inflammatory activity (IC_50_ = 4.63 ± 0.3 mg/mL) for this essential oil.

Among the various major compounds, three (linalyl acetate, menthol, and α-pinene) have been widely investigated for their anti-inflammatory effects. Regarding menthol, it was demonstrated (in vitro) in 1998 that it is able to inhibit the production of inflammation mediators by monocytes, namely interleukin-1 (IL-1), prostaglandin E_2_ (PGE_2_), and leukotriene B_4_ (LTB_4_) [58]. This was also confirmed in vivo in subsequent investigations. Indeed, in rats treated with ethanol for the induction of gastric ulcers, 50 mg/kg of menthol reduced the levels of IL-6 and TNF-α [59]. In addition, in rats given a drug containing 30–55% menthol for 60 days, a reduction in blood levels of IL-4 and IL-10 was detected [60]. In the same year, an anti-inflammatory effect was noted in rats receiving a formulation (250 mg/kg and 500 mg/kg) containing menthol, camphor, and thymol by a model of inflammation induced by turpentine [61]. Concerning α-pinene, an in vitro study carried out by Rufino et al. [62] showed that this molecule strongly inhibits the expression of catabolic and inflammatory genes, as well as catabolic and inflammatory pathways induced by IL-1β, such as the activation of NF-κB and JNK. These results have been confirmed in vivo by several researchers. Indeed, causing inflammatory responses by lipopolysaccharide (LPS) in mice, this bicyclic monoterpene was able to block the NF-κB, MAPK, and COX-2 pathways and decrease the synthesis of nitric oxide (NO), TNF-α, and IL-6 [63]. Effectively, with an inflamed hind paw model, it was found that α-pinene exhibits a potent effect on COX-2 overexpression [64].

Furthermore, Khoshnazar and colleagues were interested in the neuro-protective effect of this monoterpenoid molecule in ischemic stroke. To do this, they carried out two successive studies by inducing the occlusion of the middle cerebral artery in male rats, followed by 24-h reperfusion [65,66]. The intraperitoneal administration of α-pinene (100 mg/kg) decreased (in the striatum, cortex, and hippocampus) the concentration of IL-6, NO, and malondialdehyde (MDA) [66], as well as the expression of IL-1 and TNF-α [65]. For linalyl acetate, a remarkable reduction in carrageenan-induced edema was observed in rats at doses of 32, 64, and 96 mg/kg [67].

### 2.6. Dermatoprotective Activity

Skin aging is a natural process related to endogenous (metabolic, cellular, and hormonal processes) and exogenous (chronic exposure to pollutants, toxic chemicals, ionizing radiation, etc.) factors that cumulatively damage skin appearance and physiology [68,69]. In our study, we evaluated the dermatoprotective activity of PGEO at the three phenological stages by testing its inhibitory effect on tyrosinase, an enzyme activating tyrosine oxidation, leading to melanin secretion (Table 6). We found that at the full flowering stage, our oil displayed the best activity with an IC_50_ value of 124.49 ± 0.07 μg/mL compared to the standard, quercetin (IC_50_ = 97.29 ± 0.03 μg/mL).

Our results are in agreement with those obtained by Lohani et al. [70] and El Aanachi et al. [71], who in vitro the photoprotective activity of this plant in vitro by determining the sun protection factor (SPF), which showed values of approximately 6.45 [70] and 31.91 [71] for the essential oil and methanol extract, respectively. In addition to this potential, Lohani et al. [70] recorded an important anti-tyrosinase activity of the methanol extract (IC_50_ = 21.11 ± 0.38 μg/mL). From this, and from the antioxidant properties already confirmed in our work, it can be deduced that preparations based on *P. graveolens* can be used as sunscreen formulations for protecting the skin against sunburn and to slow down skin aging.

## 3. **Material and Methods**

### 3.1. Plant Collection and Extraction

The aerial parts of *P. graveolens*, were harvested from Sahel Boutaher (34°29′52.3″ N 4°47′11.9″ W) in the region of Taounate, Northwest Morocco. The plants were identified and confirmed by the botanists at the Botany Departement of the Scientific Institute of Rabat, Morocco. Voucher specimens of each plant were deposited in the herbarium under the voucher specimen code RAB 10672. The extraction of PGEO was conducted using hydrodistillation in a Clevenger-type apparatus(VWR, Radnor, PA, USA). Indeed, 100 g of the dry matter of the aerial parts were placed in water and brought to a boil for 3 h. The oil was recovered and then stored at a temperature of 4 °C.

### 3.2. GC-MS Analysis of Essential Oils

The chemical components of PGEO were determined using GC-MS analysis. Indeed, a Hewlett-Packard (HP6890) GC instrument (Santa Clara, CA, USA) coupled with an HP5973 MS and equipped with a 5% phenylmethyl silicone HP-5MS capillary column (30 m × 0.25 mm × film thickness 0.25 μm) was used in GC analysis. The used column temperature increased from 50 °C for 5 min to 200 °C with a 4 °C/min rate. Helium with a 1.5 mL/min flow rate and a split mode (flow: 112 mL/min, ratio: 1/74.7) was the used carrier gas. The hold time was 48 min, while the temperature of the injector and detector was 250 °C. The machine was led by a computer system type “HP ChemStation”, managing the functioning of the machine and allowing us to follow the evolution of chromatographic analyses. Diluted samples (1/20 in methanol) of 1 μL were injected manually. In addition, 70 eV ionization voltage, 230 °C ion source temperature, and 35–450 (*m/z*) scanning range were the MS operating conditions. Finally, the identification of different compounds was performed by the comparison of MS spectra with the library and matching the Kovats index (Library of NIST/EPA/NIH MASS SPECTRAL LIBRARY Version 2.0, build 1 July 2002). The quantification of the different compounds was obtained by internal normalization on the total area of peaks detected in each chromatogram.

### 3.3. Antibacterial Activity

#### 3.3.1. Bacterial Strains and Growth Conditions

The antibacterial activity was evaluated against the following six bacterial strains representing Gram-positive and Gram-negative bacteria: *Escherichia coli* ATCC 25922, *Proteus mirabilis* ATCC 25933, *Salmonella Typhimurium* ATCC 700408, *Bacillus subtilis* ATCC 6633, *Staphylococcus aureus* ATCC 29213, and *Listeria monocytogenes* ATCC 13932. The tested bacterial strains were prepared by inoculating a loopful from the frozen stock (−20 °C) in Mueller–Hinton Agar (Biokar, Beauvais, France) and incubated at 37 °C for 24 h under an aerobic environment.

#### 3.3.2. Disc Diffusion Assay

The primary screening of the antimicrobial activity of the studied samples was evaluated by the disc diffusion method according to the previously published investigation [72]. Briefly, the culture suspension of each species was inoculated in Mueller–Hinton Agar (Biolife, Italiana, Milano-Italy). Afterward, 6 mm diameter sterile paper discs soaked with 10 µL of *Pelargonium graveolens* EOs were deposited on each plate. Chloramphenicol (30 µg) was used as a positive control for bacteria strains, while dimethylsulfoxide (DMSO) (10 µL; 5%) was used as a negative control. The plates were incubated at the following growth conditions; 37 °C for 24 h, 25 °C for 48 h, and 25 °C for 72 h for bacteria, yeast, and fungi, respectively. After incubation, the inhibitory diameters were measured in millimeters, and the results were expressed as means ± Standard Deviation of three replicates.

#### 3.3.3. Determination of MIC and MBC

The minimum inhibitory concentration (MIC) and minimum bactericidal concentration (MBC) were evaluated by the broth microdilution method as reported previously by Bouyahya and his group [37]. Briefly, in a 96-well plate, 50 μL of different concentrations of *Pelargonium graveolens* essential oils (as % (*v*/*v*)) were deposed in each well. Then, 50 μL of bacteria at 10^6^ CFU/mL were added to each well, and then the plate was incubated at 37 °C for 18 h. After incubation, 10 μL of resazurin was added to each well to assess bacterial growth. The bacteria growth was revealed by detecting the reduction of blue dye resazurin to pink resorufin after 2 h of incubation at 37 °C. Thus, the MIC was determined as the lowest concentration of essential oils that have induced changes in the resazurin color. However, the MBC was determined by transferring 10 μL from the negative subcultures into Tryptone Soy Agar (Biokar, Beauvais, France) followed by incubation at 37 °C for 24 h, and the lowest concentration that did not present any growth on the media was considered as the MBC [73].

### 3.4. Antioxidant Activity

The antioxidant activity of PGEO was evaluated by using three complementary methods, DPPH, FRAP, ABTS, and H_2_O_2_ assays, according to the previously published protocols [36,74,75]. The results are expressed as the concentration of PGEO providing 50% inhibition (IC_50_) and calculated by plotting the inhibition degrees against the essential oil concentrations. Trolox and ascorbic acid were used as positive controls. The assays were carried out in triplicate, and the IC_50_ values were presented as means ± SD.

### 3.5. In Vitro Anti-Diabetic Assay

The anti-diabetic effect of the PGEO was determined by testing the ability of the oils to inhibit the enzymatic effect of α-amylase and α-glucosidase according to the same methods as our previously published study [76], and the determination of lipase inhibitory activity was according to the method described by Hu et al. [77].

### 3.6. Lipoxygenase (5-LOX) Inhibition Assay

Lipoxygenase inhibitory activity of PGEO at three phenological stages was evaluated by following the linoleic acid oxidation at 234 nm, according to the previously published method [78]. Briefly, 20 µL of oil and 20 µL of 5-LOX from Glycine max (100 U/mL) were pre-incubated with 200 µL of phosphate buffer (0.1 M, pH 9) at room temperature for 5 min. The reaction was started by the addition of 20 µL of linolenic acid (4.18 mM in ethanol) and followed for 3 min at 234 nm. The results correspond to the mean ± SEM of three independent assays, each performed in triplicate. Quercetin was used as a positive control.

### 3.7. Dermatoprotective Activity

The tyrosinase inhibitory activity was performed to evaluate the dermatoprotective effect, following the method described by [37] with some modification. Briefly, the sample (20 μL) was added to the tyrosinase solution (100 μL, 333 unit/mL in phosphate buffer 50 mM, pH 6.5) and kept at 37 °C for 10 min. Then, 200 μL of the substrates (L-DOPA, 5 mM) were added. After 30 min of incubation at 37 °C, the absorbance was determined at 510 nm using a spectrophotometer. The percent inhibition of tyrosinase activity was calculated at the concentrations of 30, 60, 90, and 120 μg/mL, and the 50% inhibitory concentrations were calculated (IC_50_). Quercetin was used as a positive control.

### 3.8. Statistical Analysis

All of the experiments were conducted in triplicate, and the obtained results are expressed as mean ± SD. The data were analyzed using SPSS software version 21(IBM SPSS statistics for Windows, Version 21.0 Armonk, NY, USA), and comparisons between means were conducted using one-way ANOVA, followed by the Tukey test. The differences between the means were considered significant when *p* < 0.05.

## 4. Conclusions and Perspectives

In the present work, the chemical composition and some of the biological properties of PGEO obtained from plant material at three phenological stages were investigated. All three EOs exhibited similar chemical composition with quantitative changes related to major constituents. PGEO showed important antibacterial, antioxidant, antidiabetic, anti-inflammatory, and dermatoprotective effects. The obtained results suggest the use of essential oils for different purposes thanks to their bioactivities, such as in the field of cosmetics or preservants. In addition, other in-depth studies should be approached to highlight the molecular mechanisms of essential oils and their major compounds for possible pharmaceutical applications. In addition, toxicological studies are also important to show the safety of the tested products.

## Figures and Tables

**Table 1:** Composition of PGEOs obtained by GC-MS analysis from plant material collected at different phenological stages, namely vegetative stage, beginning flowering stage, and full flowering stage. The values represented the average of 3 determinations.

Kovats Index (NIST)	Compounds	Phenological Stages (%)		
		Vegetative Stage	Beginning Flowering Stage	Full Flowering Stage
940	*α*-Pinene	5.72	5.64	4.28
951	Camphene	0.39	0.44	0.38
1010	*3*-Carene	3.88	3.31	1.52
1022	*δ*-Carene	0.27	0.23	0.17
1030	Limonene	0.14	Nd	nd
1030	*p*-Menthene	7.87	6.7	9.97
1039	*cis*-Ocimene	1.24	1.37	1.07
1046	1,8-Cineole	nd	0.14	nd
1050	*trans*-*β*-Ocimene	0.11	Nd	nd
1060	*cis*-Sabinene	0.2	0.16	0.17
1101	α-Thujone	0.3	0.32	0.24
1115	*trans*-Rose oxide	0.3	0.33	1.29
1134	*cis*-Rose oxide	0.11	0.12	0.59
1134	*cis*-Limonene oxide	nd	Nd	nd
1143	Camphor	0.51	0.44	0.43
1151	Citronellal	nd	Nd	0.29
1154	*p*-Menthone	0.1	Nd	0.27
1159	Isoborneol	5.19	4.02	6.96
1173	Menthol	15.8	14.06	20.57
1178	Naphthalene	0.2	0.26	0.2
1182	Isomenthol	nd	0.14	0.15
1186	Cryptone	0.18	nd	nd
1242	Z-Citral	0.67	0.41	0.52
1253	Linalyl acetate	3.38	3.9	2.97
1273	Isogeraniol	15.47	15.05	9.14
1284	*trans*-Anethole	0.12	0.15	nd
1296	Azulene	0.82	0.52	0.23
1305	*Geranyl formate*	1.56	2.06	1.65
1318	Iso-menthyl acetate	0.12	0.21	0.44
1349	α-Cubebene	0.57	0.71	0.39
1373	Isoledene	0.19	0.16	0.12
1376	α-Copaene	nd	nd	0.13
1378	Copaene	0.25	0.38	0.34
1388	*β*-Cubebene	3.46	4.25	2.05
1390	Isolongifolene	1.78	2.05	1.49
1402	Junipene	0.2	1.23	0.37
1409	*α*-Bourbonene	nd	2.71	1.72
1412	*β*-Bourbonene	1.95	nd	0.2
1423	*trans*-Caryophyllene	0.97	1.05	0.98
1426	Calarene	0.42	0.41	0.37
1431	Aromadendrene	1.05	1.48	1.53
1442	Dehydroaromadendrene	1.07	0.55	0.51
1444	*α*-Guaiene	0.88	1.06	1.2
1451	Germacrene-D	0.23	0.23	0.13
1456	*α*-Caryophyllene	0.42	0.35	0.32
1456	Seychellene	0.39	0.76	0.42
1457	*α*-Humulene	0.57	0.67	0.4
1480	*Ω*-Cadinene	1.06	1.16	0.98
1482	Ledene	0.44	0.61	0.82
1491	Valencene	0.63	0.82	0.83
1500	*ẟ*-Himachalene	nd	nd	0.13
1503	Eremophilene	8.34	9.02	8.19
1511	*β*-bisabolene	0.54	0.53	0.49
1537	Elemol	1.86	2.3	2.32
1541	*α*-calacorene	nd	nd	0.14
1618	*trans*-Longipinocarveol	nd	0.25	0.6
1643	Cubenol	0.3	0.35	0.46
1890	Ledene oxide	0.17	0.28	0.18
2038	Humulene oxide	0.27	0.28	1.2
	Octahydro-naphthalene	0.51	0.39	1.01
	Phenyl tiglate	1.67	1.64	1.97
	**Total identified compounds%**	92.98	92.89	93.02
	**Monoterpene hydrocarbons%**	20.84	18.63	17.99
	**Oxygenated monoterpenes %**	39.08	35.1	41.31
	**Sesquiterpenes hydrocarbons%**	25.41	30.19	24.25

nd: not detected.

**Table 2:** The inhibitory diameters (mm) of PGEO at three developmental stages against bacterial strains.

Microorganisms/Gram (+ or −)	*P. graveolens* EOs			Chloramphenicol (30 µg)
	Vegetative Stage	Beginning Flowering Stage	Full Flowering Stage	
*Escherchia coli* ATCC 25922 (−)	11.00 ± 0.17	12.47 ± 0.06	13.43 ± 0.21	22.47 ± 0.21
*Proteus mirabilis* ATCC 25933 (−)	11.03 ± 0.11	12.73 ± 0.15	14.57 ± 0.21	21.27 ± 0.21
*Salmonella Typhimurium* ATCC700408 (−)	8.27 ± 0.11	9.93 ± 0.15	11.10 ± 0.20	13.17 ± 0.25
*Bacillus subtilis* ATCC 6633 (+)	14.13 ± 0.11	15.23 ± 0.15	16.17 ± 0.15	15.87 ± 0.21
*Staphylococus aureus* ATCC 29213 (+)	14.97 ± 0.15	16.10 ± 0.20	16. 53 ± 0.15	26. 53 ± 0.30
*Listeria monocytogenes* ATCC 13932 (+)	15.23 ± 0.15	16.83 ± 0.25	17.30 ± 0.17	29.07 ± 0.58

Zones of inhibition included the 6 mm diameter well, at a concentration of 50 μL of oil/well. Diameters in mm. *P. graveolens*: *Pelargonium graveolens*; EOs: essential oils; −: Gram-negative bacteria; +: Gram-positive bacteria.

**Table 3:** MIC and MBC measured of PGEOs at three developmental stages.

Microorganisms/Gram (+ or −)	*P. graveolens* EOs in % (*v*/*v*)						Chloramphenicol (µg/mL)
	Vegetative Stage		Beginning Flowering Stage		Full Flowering Stage		
	MIC	MBC	MIC	MBC	MIC	MBC	
*Escherchia coli* ATCC 25922 (−)	1	2	1	2	0.5	1	4
*Proteus mirabilis* ATCC 25933 (−)	2	2	1	2	1	1	4
*Salmonella Typhimurium* ATCC700408 (−)	2	4	2	4	2	2	64
*Bacillus subtilis* ATCC 6633 (+)	0.5	0.5	0.25	0.5	0.25	0.5	32
*Staphylococus aureus* ATCC 29213 (+)	0.5	0.5	0.25	0.5	0.25	0.5	4
*Listeria monocytogenes* ATCC 13932 (+)	0.5	0.5	0.12	0.5	0.25	0.25	2

MIC: minimal inhibitory concentration; MBC: minimal bactericidal concentration; *P. graveolens*: *Pelargonium graveolens*; EOs: essential oils.

**Table 4:** The antioxidant activity of P. graveolens EOs expressed as IC_50_.

Method	*P. graveolens* EOs			Positive Controls	
	Vegetative Stage	Beginning Flowering Stage	Full Flowering Stage	Ascorbic Acid	Trolox
***DPPH***	138.23 ± 0.16 ^c^	119.49 ± 2.01 ^d^	83.26 ± 0.01 ^e^	15.13 ± 0.04 ^a^	24.31 ± 0.02 ^b^
***FRAP***	151.21 ± 0.08 ^c^	139.35 ± 0.12 ^d^	116.42 ± 0.07 ^e^	31.47 ± 1.07 ^a^	48.25 ± 0.05 ^b^
***ABTS***	174.95 ± 1.14 ^c^	153.09 ± 0.05 ^d^	132.25 ± 0.11 ^e^	42.22 ± 0.05 ^a^	61.48 ± 1.32 ^b^
***H*_2_ *O*_2_**	77.35 ± 0.04 ^b^	64.81 ± 1.14 ^c^	48.67 ± 0.04 ^d^	23.34 ± 0.03 ^a^	-

Different letters indicate significant differences (*p* < 0.05; n = 3). *P. graveolens*: *Pelargonium graveolens*; EOs: essential oils; *DPPH*: 1,1-diphenyl-2-picrylhydrazyl; *FRAP*: ferric reducing antioxidant power; *ABTS*: 3 ethylbenzothiazoline 6-sulfonate; *H*_2_*O*_2_: hydrogen peroxide.

**Table 5:** The antidiabetic activity of PGEOs (IC_50_ in μg/mL) at three developmental stages. Results are presented as means ± SD (standard deviations) for triplicate assays.

Assays	*Pelargonium graveolens* EOs			Positive Controls	
	Vegetative Stage	Beginning Flowering Stage	Full Flowering Stage	Acarbose	Orlistat
α-Amylase IC_50_ (µg/mL)	95.24 ± 0.03 ^a^	69.31 ± 0.08 ^b^	43.33 ± 0.01 ^c^	27.21± 0.08 ^d^	-
α-Glucosidase IC_50_ (µg/mL)	49.21 ± 0.05 ^a^	26.37 ± 0.02 ^b^	19.04 ± 0.01 ^c^	10.01 ± 0.03 ^d^	-
Lipase IC_50_ (µg/mL)	63.27 ± 0.01 ^a^	47.62 ± 1.03 ^b^	24.33 ± 0.05 ^c^	-	18.27 ± 0.03 ^d^

Different letters indicate significant differences (*p* < 0.05; n = 3), (-): not tested.

**Table 6:** In vitro anti-inflammatory and dermatoprotective activities of PGEOs at three phenological stages.

Assays	*Pelargonium graveolens* EOs			Positive Control
	Vegetative Stage	Beginning Flowering Stage	Full Flowering Stage	Quercetin
5-Lipoxygenase	84.21 ± 0.08 ^a^	61.54 ± 0.07 ^b^	39.31 ± 0.01 ^c^	24.89 ± 0.02 ^d^
Tyrosinase	187.29 ± 0.03 ^a^	152.39 ± 0.02 ^b^	124.49 ± 0.07 ^c^	97.29 ± 0.03 ^d^

Different letters indicate significant differences (*p* < 0.05; n = 3).

## Data Availability

Not applicable.
